# Single cell RNA sequencing sheds light on infiltrating T cells in idiopathic inflammatory myopathies

**DOI:** 10.15252/emmm.202318190

**Published:** 2023-09-28

**Authors:** Momina Yazdani, Lukas Mach, Michela Noseda

**Affiliations:** ^1^ National Heart and Lung Institute Imperial College London London UK

**Keywords:** Chromatin, Transcription & Genomics, Immunology, Musculoskeletal System

## Abstract

Idiopathic inflammatory myopathies (IIM), also referred to as “myositis,” are a group of heterogeneous autoimmune disorders characterised by muscle weakness, atrophy and progressive reduced mobility (Lundberg *et al*, 2021). IIM represent a significant health burden in adult populations, affecting individuals at a mean age of 50 with an estimated prevalence of 2.9–34 per 100,000 (Dobloug *et al*, 2015; Svensson *et al*, 2017). IIM encompass several subtypes including dermatomyositis, immune‐mediated necrotising myopathy, inclusion‐body myositis, antisynthetase syndrome and polymyositis, which are characterised by specific clinical features, histopathological findings and autoantibody status (Pinal‐Fernandez *et al*, 2020).

## Graphical abstract

M. Noseda, M. Yazdani and L. Mach discuss the study by A. Argyriou *et al*, in this issue of EMBO Mol. Med., that identifies a muscle tissue resident memory T‐cell signature in patients with idiopathic inflammatory myopathies (IIM) and a transcriptomic map to identify novel therapeutic targets in IIM.

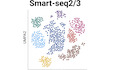

Idiopathic inflammatory myopathies can be challenging to manage owing to the variety of subtypes, often indolent presentation, variable autoantibodies and heterogeneous multiorgan involvement, which contribute to the limited understanding of their pathogenesis (Selva‐O'Callaghan *et al*, 2018). Current first‐line treatment still consists of broad immunosuppressive therapies including corticosteroids, azathioprine and methotrexate, which carry significant side effects and impact quality of life (Lundberg *et al*, 2021). Partial response to treatment can result in disease flares, and although clinical studies are scarce, epidemiological data suggest that up to 80% of patients can suffer from recurrent acute episodes or experience chronic, continuous symptoms (Bronner *et al*, 2006). Therefore, the search for more precise and effective immunomodulatory treatments as well as improved risk stratification and predictors of relapse in IIM remains a high priority.

Single cell RNA‐sequencing (scRNA‐seq) has already furthered our understanding of some inflammatory and autoimmune conditions. The recent identification of CD8^+^ T cells specific for the autoantigen α‐myosin in Immune Check Point Inhibitor Myocarditis showcases the power of this technology to yield new insights into disease pathogenesis (Axelrod *et al*, 2022). In IIM, previous studies using bulk RNA sequencing suggested that activation of Type 1 and 2 interferon pathways, which may be therapeutically targeted, differs in myopathy subtypes (Morand *et al*, 2019; Pinal‐Fernandez *et al*, 2019). However, further granularity at single cell resolution is now needed to identify the responsible immune cell populations, draw mechanistic inferences, define disease signatures at subtype level and design novel effective therapies.

In this issue of *EMBO Mol Med*, Argyriou *et al* (2023) exemplify the power of applying leading edge technologies including scRNA‐seq paired with flow cytometry to deeply profile the muscle resident and peripheral blood (PB) T‐cell populations from patients affected by IIM. Harnessing Smart‐seq2/3, a scRNA‐seq technology that allows long read sequencing, the authors could perform deep immunophenotyping to analyse key T‐cell receptors (Miranda *et al*, 2023). Through an unbiased clustering approach of 1,402 muscle T cells and 1,417 PB memory T cells, across six patients with IIM and one undefined myopathy, the authors defined the landscape of muscle infiltrating T cells in IIM (Fig 1).

**Figure 1:**
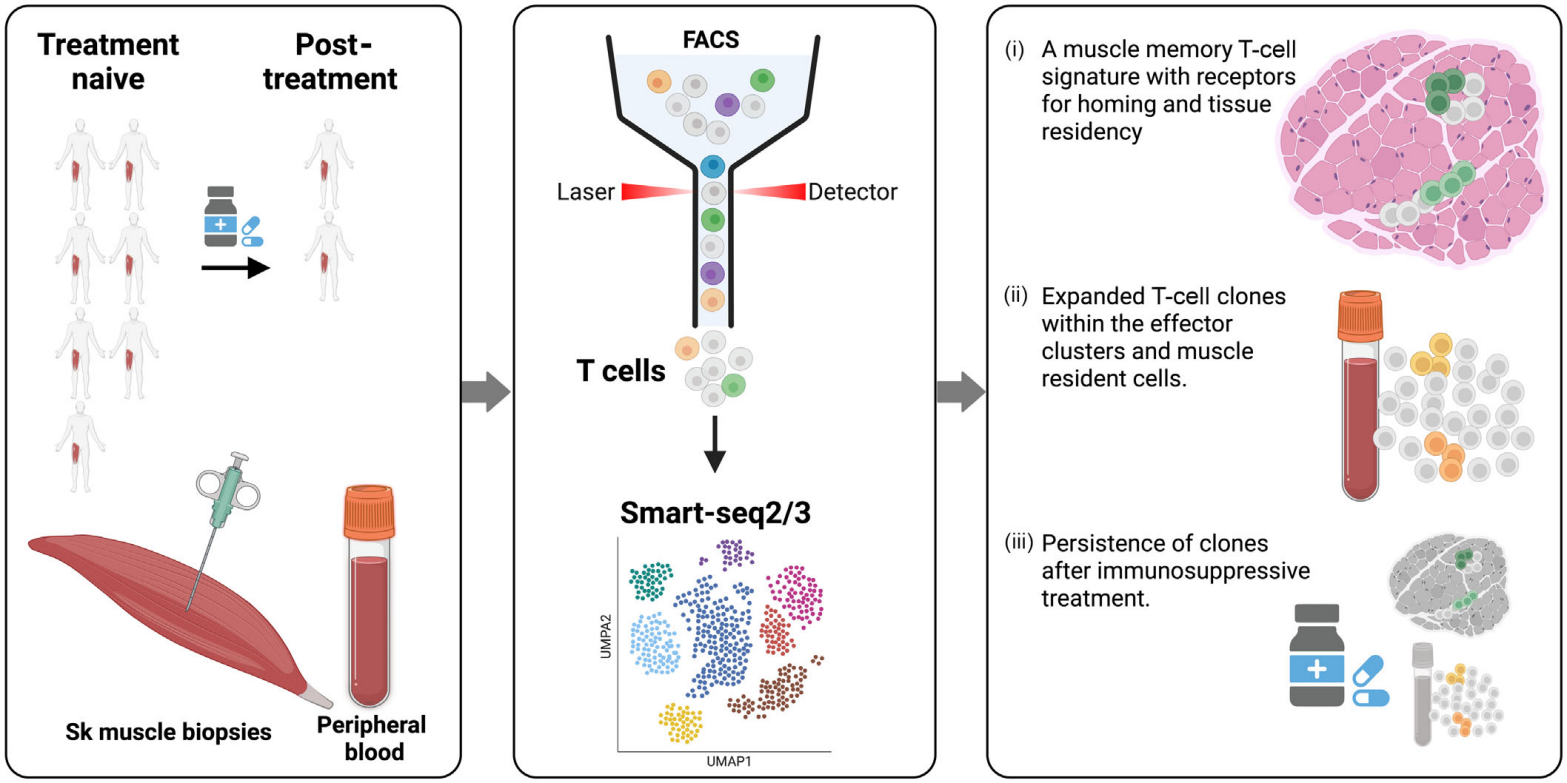
Single cell RNA sequencing of peripheral and infiltrating T cells in myositis (A) Argyriou *et al* (2023) recruited seven patients with inflammatory myopathies obtaining skeletal muscle biopsies and peripheral blood (PB) mononucleated cells. T‐cell muscle infiltration was confirmed with CD3 immunostaining. Two patients underwent tissue biopsy and PB mononucleated cells isolation after treatment. (B) Tissue infiltrating and PB T‐cells were isolated and sorted using FACS. Smart‐seq2/3 allowed transcriptome sequencing of 1,402 cells in biopsies and 1,417 cells from PB. Unbiased clustering and Uniform Manifold Approximation and Projection (UMAP) embedding identified central memory (CM) cells, CZMB^+^ effector memory (EM) cells, HOBIT^+^ tissue resident memory (TRM), EGR‐1^+^ TRM cells, HLA‐DR^+^ TRM cells, KLRB1^+^ EM cells, CD69^+^ EM cells, CXCL 13^+^ TRM cells, Regulatory T cells (Tregs) and proliferating T cells. (C) The key findings included identification of muscle resident memory T cells, clonal expansion of tissue resident and PB T cells and persistence of the identical clones in blood and tissue after immunosuppressive therapy (Figure was created using Biorender).

They demonstrate that cytotoxic, effector memory, tissue‐resident, proliferating and regulatory T cells are all present within the skeletal muscle of IIM patients. Populations of effector T memory cells are seen across all phenotypes of IIM with distinct gene expression profiles observed in specific subtypes. However, the interpretation of these results is limited by the modest sample size as tissue resident T cells could only be isolated from half of the initial cohort, which restricts our appreciation of their role to a subset of IIM patients.

Clonally expanded T effector and tissue resident T cells were identified by analysing T‐cell receptor alpha and beta chains,  an established method for identifying T‐cell clonality. New approaches demonstrated that tracking somatic mutations in mitochondrial DNA is a more accurate and higher throughput method to define clonality in a variety of cell types, beyond T cells, and would be interesting to validate in IIM (Lareau *et al*, 2023).

Overall, these findings give early granularity into the specific T‐cell populations in IIM and may propel the discovery of targeted therapies based on disease‐specific T‐cell populations.

In parallel, T‐cell populations in the PB of patients with IIM are composed of central and effector memory T cells and NK‐like CD8^+^ T cells. The latter cytotoxic T‐cell population was previously reported in other diseases such as coeliac disease, suggesting a shared mechanism of dysregulation across several immune‐mediated diseases (Meresse *et al*, 2006).

Finally, persistent T‐cell clones were detected at 9‐month follow‐up despite resolution of infiltrates on muscle biopsy and improvement in classic serological markers in two patients. It remains to be understood whether this pool of cells is indicative of incomplete response to therapy and whether targeting these clones could prevent reactivation of disease.

In this study, the authors have elegantly explored the T‐cell architecture in both muscle and circulating blood, identifying tissue‐resident cell clusters and disease‐specific signatures in IIM. In the long term, it could lead to identification of novel targets for treatment, with the aim of designing specific drugs to target pathogenic infiltrating muscle T cells.

This work provides a first exploration of T‐cell infiltration in IIM using scRNA‐seq. Single cell sorting and sequencing proves to be a sensitive approach to detect and analyse rare T cells and has potential for stratification of IIM patients. A larger number of patients will be required to further study differences across IIM subtypes and autoantibody status and, classify the diverse mechanisms driving the heterogeneity of IIM (Pinal‐Fernandez *et al*, 2020).

Comparison with T cells from healthy controls is a challenging task, as T cells are rare in healthy skeletal muscle. Here, single cell sequencing was performed on memory T cells from PB of healthy donors treated both with and without collagenase. Interestingly, enzymatic treatment appeared to have an impact on gene expression, providing a control for this technical variable. In future, integration of data with publicly available data sets including those made available via the Human Cell Atlas (https://www.humancellatlas.org/) as reference will strengthen comparative studies across healthy and diseased populations, ancestries, sexes and other variables (Miranda *et al*, 2023).

Going forward, the role of other immune and inflammatory cell populations in IIM, such as macrophages, should be explored. Expanding the use of single cell transcriptomic and multiomic analyses, including spatial transcriptomics, to study the immune cellular compartment in patients with IMM will provide novel insights into phenotype‐specific disease pathogenesis and pave the way towards new precision medicine treatments.
